# A Portable Arsenic Sensor Integrating *Bacillus megaterium* with CMOS Technology

**DOI:** 10.1021/acssynbio.4c00895

**Published:** 2025-04-11

**Authors:** Chelsea Y. Hu, John McManus, Fatemeh Aghlmand, Tracy Mei, Elin Larsson, Azita Emami, Richard M. Murray

**Affiliations:** † Division of Biology and Bioengineering, California Institute of Technology, Pasadena, California 91125, United States; ‡ Division of Engineering and Applied Science, California Institute of Technology, Pasadena, California 91125, United States; § Department of Chemical Engineering, Texas A&M University, College Station, Texas 77843, United States

**Keywords:** whole-cell sensor, bacillus megaterium, cmos, integrated biosensor, spores, arsenic detection

## Abstract

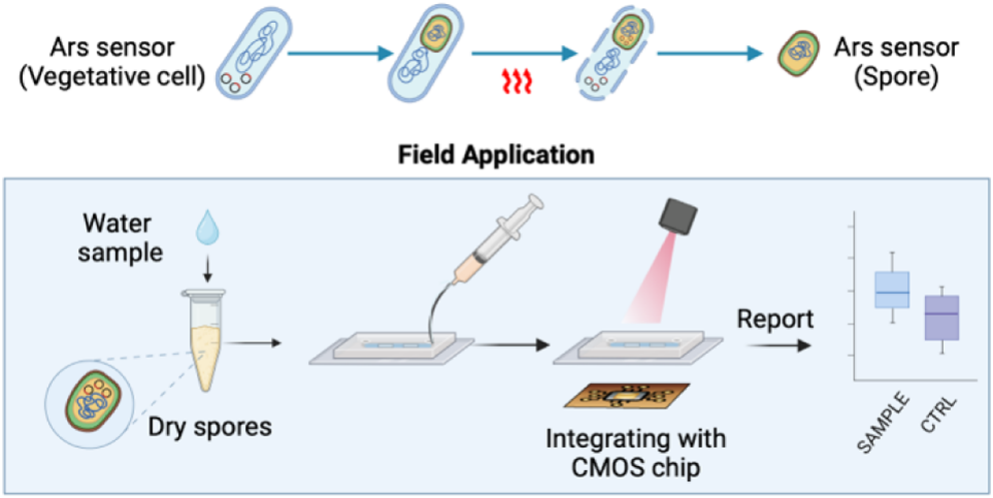

Bacteria innately monitor their environment by dynamically regulating gene expression to respond to fluctuating conditions. Through synthetic biology, we can harness this natural capability to design cell-based sensors. *Bacillus megaterium*, a soil bacterium, stands out due to its remarkable heavy metal tolerance and sporulation ability, making it an ideal candidate for heavy metal detection with low transportation costs. However, challenges persist: the synthetic biology toolkit for this strain is underdeveloped, and conventional whole-cell sensors necessitate specialized laboratory equipment to read the output. In our study, we have genetically modified *B. megaterium* for arsenic detection and established a detection threshold below the EPA’s recommendation of 10 ppb for drinking water in both vegetative and spore forms. Additionally, we have integrated both engineered *B. megaterium* living cells and spores with a complementary metal-oxide-semiconductor (CMOS) chip, providing a proof-of-concept for field-deployable arsenic detection. We show that the limit of detection (LOD) of our integrated sensor is within the range to test arsenic levels in soil and food. As a proof of concept, this work paves the way for the deployment of our sensor in resource-limited settings, ensuring real-time arsenic detection in challenging environments.

## Introduction

Arsenic is a naturally occurring element widely distributed in the Earth’s crust, with significant presence in groundwater, soil, crops, and various minerals. Chronic exposure to arsenic, even at low levels, poses severe health risks including skin lesions, cardiovascular diseases, and various forms of cancer.^1,2^ The US Environmental Protection Agency (EPA) has set the maximum contaminant level (MCL) for arsenic in drinking water at 10 parts per billion (ppb) or 0.13 μM; the EPA also set the soil screening level (SSL) for residential areas at 0.39 mg/kg (approximately 390 ppb).^3^ The Occupational Safety and Health Administration (OSHA) has established an arsenic permissible exposure limit (PEL) of 10 μg/m^3^ in air (approximately 0.01 ppb) over an 8-h workday,^4^ and the US Food and Drug Administration (FDA) has set an action level for rice cereals for infants at 100 ppb.^5^ The risks to human health and known permissible arsenic levels underscores the importance of monitoring arsenic concentrations in the environment. Traditional methods for arsenic detection, such as atomic absorption spectroscopy (AAS) and inductively coupled plasma mass spectrometry (ICP-MS), have historically offered high sensitivity and accuracy but are often limited by their high cost, complex sample preparation, and requirement for sophisticated laboratory infrastructure. Conversely, modern arsenic field test methods face limitations due to the unreliability of low-cost kits, which have been reported to produce false positive results.^6^ These limitations underscore the need for innovative, cost-effective, and portable solutions for real-time arsenic monitoring in diverse environmental field settings.

Whole-cell biosensors are a type of sensor that detects environmental contaminants using living cells. These sensors produce measurable signals when exposed to target substances, taking advantage of the natural cellular response to chemical stimuli. *Escherichia coli* (*E. coli*) is one of the most extensively engineered organisms for whole-cell arsenic sensors. Over the past decade, significant efforts have been made to improve the sensitivity of *E. coli*-based arsenic sensors, with many achieving remarkable detection limits below 10 ppb in water.^7−15^ However, whole-cell biosensors, despite their low cost and remarkable detection capabilities, are not yet widely used in practice. A major limitation is the challenge of transporting live bacterial cells for end point testing in remote settings, as this requires a cold chain to maintain viability. In laboratory settings, where transportation is not a concern, more precise methods like AAS and ICP-MS are typically preferred, further limiting the adoption of whole-cell biosensors. Consequently, the requirement for meticulous maintenance of live cells significantly restricts the practical applicability of whole-cell biosensors and increases their overall cost. This highlights the need for developing whole-cell biosensors with minimal maintenance requirements and lower operational costs. Additionally, while reliable detection of low arsenic concentrations is critical, the sensor’s detection range is equally vital for broader applicability. The detection range refers to the concentration span over which the sensor’s output signal reliably increases with the input concentration. *E. coli* has a limited detection range, between 10 and 500 ppb,^10^ constrained by arsenic’s toxic effects on the cells. The LC50 (50% lethal concentration) for arsenic in *E. coli* ranges from 200 to 2,000 ppb.^16^ Notably, most regulatory guidelines permit arsenic concentrations in soil that exceed 200 ppb. Therefore, for the sensors to effectively detect arsenic, the chassis must tolerate concentrations well beyond the maximum allowable levels, ensuring a relevant and practical detection range.

The Gram-positive genus *Bacillus* is known for its high resistance to environmental stressors. *Bacillus* species produce endospores under harsh conditions as part of their survival strategy. These endospores exhibit a high tolerance to various environmental challenges represent a metabolically dormant form of the cells. Spores can remain dormant for extended periods and later germinate into vegetative cells when conditions become favorable. Previous studies have shown engineered whole-cell biosensors can be preserved in spore form for up to two years in room temperature without losing functionality.^17,18^ Specifically, Valenzuela-García et al. demonstrated that an engineered arsenic biosensor in *Bacillus subtilis* (*B. subtilis*) spores remains functional after germination. Although no statistical significance was computed, the study has reported a LOD of 0.077 μM (5.9 ppb) in vegetative cells and 0.1 μM (7.7 ppb) in germinated spores.^19^ These findings highlight the potential of using spores as whole-cell sensors, which eliminates the need for cold-chain storage and greatly enhances the practicality of these biosensors for environmental monitoring.

However, *B. subtilis* is not the most robust strain within the *Bacillus* genus. *Bacillus megaterium*, a ubiquitous soil bacterium, offers additional advantages for applications beyond the laboratory due to its resilience and adaptability. Along with its spore-forming capability and widespread environmental presence, *B. megaterium* possesses several traits advantageous for biosensor development. Its larger cell size allows for higher gene expression and greater metabolic load capacity, both of which are critical for biosensor efficacy.^20,21^ Moreover, *B. megaterium* exhibits strong resistance to toxic substances, including arsenic.^22^*B. megaterium* remains relatively under-characterized as a synthetic biology chassis despite its advantages. In this study, we have leveraged the robust sensing capabilities of *B. megaterium* to enhance its potential as a field-deployable whole-cell biosensor. We hypothesize that its inherent characteristics will enable the direct use of spores for sensing, eliminating the need for germination.

CMOS technology has a wide range of applications, traditional CMOS chips are limited in their capacity to detect specific analytes like arsenic. While whole-cell sensors can detect a broad range of analytes, they often rely on sophisticated instruments to relay fluorescent signals. We have recently developed a CMOS chip capable of detecting strong fluorescent signals from living cells, laying the groundwork for seamless integration of biological and electronic systems. While the ability to achieve continuous signal transduction—translating analyte concentrations into proportional electronic signals—has yet to be fully realized, this development represents a significant step toward that goal.^23^ With its versatility, compact size (2 mm by 1.5 mm), and low cost (<$1), CMOS technology is an ideal candidate for field-deployable sensors. The integration of CMOS with whole-cell sensors leverages the sensitivity and robustness of living cells, paired with the practicality of CMOS technology, to pave the way for robust, field-ready biosensors.

In this study, we engineered an integrated arsenic sensor for field application by combining whole-cell *B. megaterium* with our customized CMOS chip. This system combines robust characteristics of *B. megaterium* and the portability of the CMOS chip, to achieve arsenic detection. To this end, we constructed an arsenic-sensing gene circuit and transformed into *B. megaterium*. Here, we demonstrate the statistically significant LOD for arsenic at 0.01 μM (0.77 ppb) in vegetative cells and 0.05 μM (3.85 ppb) in spores. These detection limits are below the EPA’s maximum contaminant level for arsenic in drinking water of 10 ppb (0.13 μM). In the integrated sensor, we show that the CMOS chip enables a proportional signal relay, translating varying fluorescent signals from *B. megaterium*—in response to arsenic concentrations—into corresponding electronic signals. The LODs achieved were 0.3 μM (23.1 ppb) for vegetative cells and 1.0 μM (77 ppb) for spores with statistical significance. These detection limits highlight the system’s potential for monitoring inorganic arsenic concentrations in soil and air samples.

## Results and Discussion

### Design and Characterization of Whole-Cell Arsenic Biosensor in *Bacillus megaterium*

**Figure 1:**
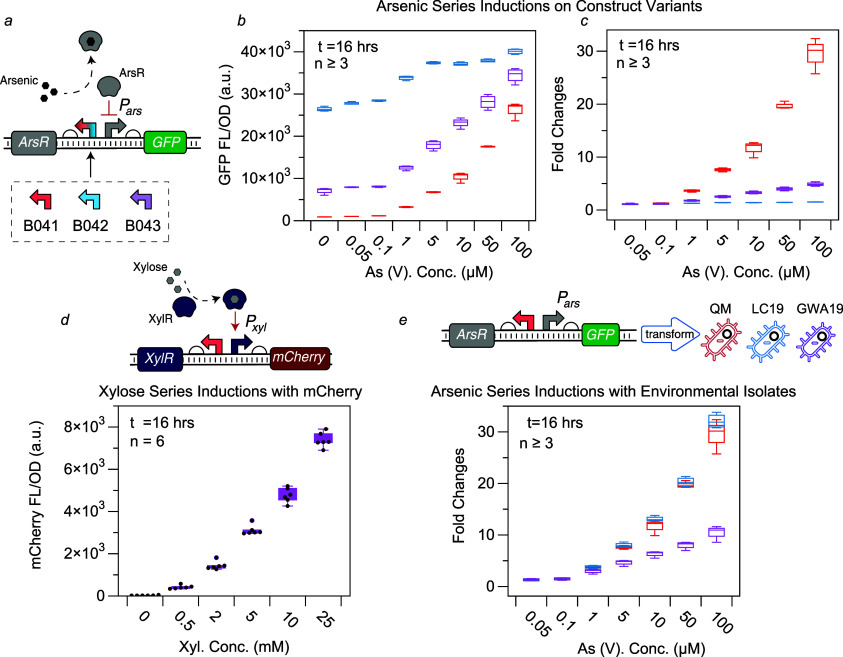
Design and characterization of a whole-cell arsenic sensor in *Bacillus megaterium*. (a) Circuit design: the arsenic sensor utilizes the *ars* operon and GFP. Transcription regulator ArsR binds to the *P_ars_* promoter, repressing downstream transcription. This repression is relieved in the presence of arsenic, and GFP is expressed. Three versions of constructs, each with a different constitutive promoter driving *ArsR*, are characterized. (b) Sensor induction: the sensor was induced with As(V) ranging from 0.05 μM to 100 μM. The response curves for the three versions of constructs, colored in red (B041), blue (B042), and purple (B043), each with a different constitutive promoter driving *ArsR*. (c) Normalized response: the same experiment as plotted in (b), with all values normalized to the none-induced conduction (0 μM) to highlight fold activation. (d) Characterization of mCherry: The mCherry fluorescent protein was characterized, with induction by xylose concentrations ranging from 0.5 mM to 25 mM. The constitutively expressed XylR binds to xylose to activate the expression of mCherry. (e) Arsenic series inductions in environmental isolates: The B041 construct was transformed into two environmental isolates of *B. megaterium*; *B. megaterium* LC19, and *B. megaterium* GWA19, along with the model strain *B. megaterium* QM, As(V) concentration ranged from 0.05 μM to 100 μM, showing sensor functionality across strains. The number of biological replicates (n) used in each experiment is indicated in all plots.

We designed the arsenic sensor utilizing the *ars* operon, which is known for its role in conferring arsenic resistance to *Bacillusspp.*(24) Within this operon, the ArsR protein acts as a repressor, binding to the promoter *P*_*ars*_ to inhibit transcription. Exposure to arsenic induces a conformational change in ArsR, leading to its detachment from DNA and thus initiating transcription.^25^ As illustrated in Figure 1a, our design features constitutive expression of the ArsR gene, while the Pars promoter regulates the transcription of a green fluorescent protein (GFP), a well-established fluorescent reporter in *B. megaterium*.^26^ The genetic circuit was cloned into the pMM1522 plasmid backbone for transformation. In this system, the presence of arsenic triggers GFP expression, providing a fluorescent signal indicative of the metalloid’s presence.

We first aimed to optimize the dynamic range of the arsenic sensor by varying the expression strength of the transcription regulator ArsR. To do this, we constructed three sensor variants, each utilizing a different constitutive promoter (B041-Pyknw, B042-yngC, and B043-metA), all derived from native promoters found in *B. subtilis*.^7^ Through titration experiments (shown in Figure 1b,c), we assessed the influence of the constitutively promoters on sensor functionality. We observed that B042 (blue), which utilizes the weakest promoter for ArsR expression, exhibited significant leaky GFP expression, whereas B041 (red), which uses the strongest promoter for ArsR expression, showed minimal GFP leakage (Figures 1b, S1a). These results align with the mechanistic role of ArsR as a transcriptional repressor; weak ArsR expression leads to incomplete repression of the *P*_*ars*_ promoter. To assess dynamic range, we calculated the ratio of the highest average induced signal to the baseline uninduced signal. B041 (red) demonstrated a 30-fold activation upon arsenic induction. Based on these findings, we selected the P_yknw_ promoter for further development.

Concurrently, we aimed to confirm that *B. megaterium* can operate in conditions that are compatible with the CMOS chip. A critical component that requires confirmation is the fluorescent protein. The CMOS chip has a detection range of 600–700 nm, beyond the detection range of GFP but within the range of mCherry and LSSmOrange. Our previous studies have shown that the CMOS chip provides a robust signal readout when detecting mCherry and LSSmOrange^27^ fluorescence expressed in *E. coli*, motivating us to test the expression and response dynamics of these two fluorescent proteins in *B. megaterium*. Since these fluorescent proteins were never tested in *B. megaterium* before, we utilized the *xyl* operon, previously shown to be functional in *B. megaterium*,^21^ to drive their expressions for characterization of these fluorecent proteins. This system employs the transcriptional regulator XylR, which activates the *P*_*xyl*_ promoter in the presence of xylose. Upon induction with xylose, mCherry expression was observed, with fluorescence signals clearly differentiating across a range of xylose concentrations from 0 to 25 mM, as shown in Figure 1d. In contrast, LSSmOrange did not exhibit any fluorescent signal (data not shown). These results confirm that mCherry can be effectively expressed in *B. megaterium* and responds with a positive correlation between fluorescence signal and xylose levels. Additionally, we tested induction under both Lysogeny Broth (LB) and M9 minimal media supplemented with 1% glucose conditions, as shown in Figure S1b and c. Although *B. megaterium* was able to grow in M9 medium, with a reduced final optical density at 600 nm (OD_600_), it did not express any fluorescent protein upon induction in this medium. This could be a result of the presence of glucose interfering with the *xyl* operon.^28^ Therefore, for the remainder of the project, we adopted LB medium for both cell culture and sensor induction.

Using a modified transformation procedure (detailed in the Supporting Information) based on the protocol published by Vorobjeva et al.,^29^ we transformed our best-performing construct, B041 (highlighted in red in Figure 1b and c), into two additional environmental isolates of *B. megaterium*. As shown in Figure 1e, both *B. megaterium* LC 19 and *B. megaterium* GWA19, along with *B. megaterium* QM, were successfully transformed with our plasmid hosted by the pMM1522 backbone. The arsenic sensor was functional in all three strains. However, the sensor transformed into *B. megaterium* GWA19 exhibited a reduced fold activation, indicating strain-to-strain differences in the whole-cell sensor function.

### Characterization of Arsenic Whole-Cell Biosensor in Vegetative *Bacillus megaterium*

**Figure 2:**
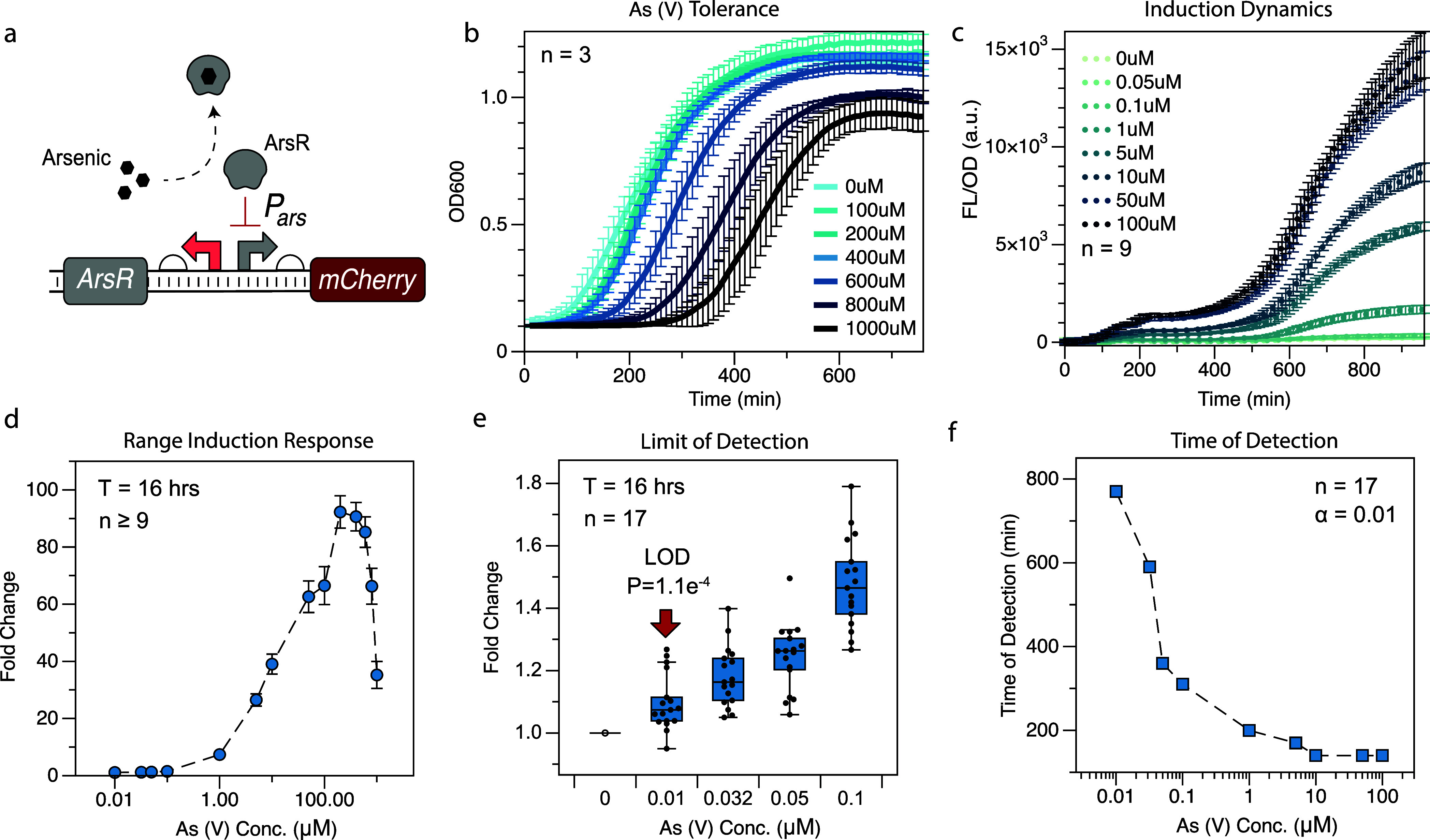
Sensor performance in vegetative *B. megaterium*. (a) Sensor construct design. The design of the arsenic sensor construct derived from B041, shown in Figure 1a, using mCherry as the reporter (b) Growth dynamic in high arsenic concentrations: Growth dynamics of B. megaterium in the presence of As(V). ranging from 100 μM to 1000 μM, over a 16-h period. (c) Fluorescent signal dynamics: mCherry fluorescence normalized by OD_600_ over 16 h, following induction at time = 0 min. (d) The sensor response to arsenic concentrations: Sensor response to As(V) ranged from 0.05 μM to 1 mM, normalized to the 0 μM induction culture, measured after 16 h postinduction. (e) Limit of detection: induction response of the sensor to low-range As(V) concentrations, spanning from 0.01 μM to 0.1 μM. The plot shows statistical significance in sensor activation with as low as 0.01 μM of As(V). (f) Time of detection. The time required for the whole-cell sensor to register a positive detection of arsenic, ranging from 0.01 μM to 100 μM, using a two-tailed *t* test with a significance threshold of 0.01. The number of biological replicates (*n*) used in each experiment is indicated in all plots.

Having separately characterized the performance of the arsenic sensor and the CMOS chip-compatible fluorescent protein mCherry, we integrated these components into our final design, as shown in Figure 2a. This design retains the genetic context from B041, using a constitutive *P_yknw_* promoter to drive the expression of ArsR, with mCherry replacing GFP as the reporter.

First, we assessed the arsenic tolerance of *B. megaterium*. Literature has established that the *Bacillusspp*. exhibit remarkable resilience to arsenic due to the *ars* operon compared to other genuses.^24^ We compared the arsenic tolerance of wildtype *B. megaterium* QM with wildtype *B. subtilis* in an As(V) series titration up to 5 mM. Our results show that 2 mM and 5 mM of As(V) induces 50% and 80% cell death in *B. subtilis*, respectively. In contrast, *B. megaterium* QM does not exhibit detectable cell death even at 5 mM of arsenic(V) (Figure S1d,1e), confirming our hypothesis that *B. megaterium* is far more resistant to arsenic than *B. subtilis*. Subsequently, we investigated the growth rates of our engineered strain in the presence of As(V). As shown in Figure 2b, we compared the growth curves of the sensor under high concentrations of arsenic(V), ranging from 100 μM to 1 mM, over 16 h. All cultures were able to grow, despite variations in maximum OD and prolonged lag phases as the concentration increased. Notably, no significant change in growth rate was observed at 100 μM, 200 μM, and 400 μM of As(V) compared to the uninduced culture, suggesting that the presence of As(V) has little impact on the intrinsic metabolism of *B. megaterium* at concentrations up to 400 μM.

Next, we conducted a low-concentration range titration from 0.05 μM to 100 μM of arsenic. Figure 2c presents a full 16-h time course of the experiment. After 16 h, we analyzed the fold changes in fluorescence relative to the uninduced culture across all arsenic concentrations. As depicted in Figure 2d, mCherry expression increased with arsenic concentrations up to 200 μM. Beyond 400 μM, the fluorescence reached a plateau and then started to decline, which aligns with our findings in Figure 2b, where cell growth was impaired starting at 400 μM, indicating that both recombinant gene expression and cell growth are impaired at this concentration.

To establish the limit of arsenic detection at low concentrations, we interrogated the detection threshold. The fold change in fluorescence of 17 independent colonies exposed to low arsenic doses (Figure 2e), ranging from 0.01 μM to 0.1 μM, reveals a detection limit as low as 0.01 μM (0.77 ppb) after 16 h, with statistical significance. In Figure 2f, we plotted the time required for each culture to register a positive detection of arsenic. This was determined by identifying the earliest time point that passed the two-tailed *t* test with significance threshold of 0.01. The results indicated that at higher concentrations (10 μM, 50 μM, and 100 μM), signals can be detected as early as 140 min; but it would take over 12 h (770 min) to detect the ultralow 0.01 μM of arsenic. The extended detection time is also likely influenced by the slow maturation of mCherry, which could take up to 114 min.^30^ Our reported LOD represents the lowest value achieved among arsenic whole cell biosensors, surpassing other systems, including *E. coli* based fluorescent sensors reported with limits at 2.3 ppb^31^ and 5.4 ppb,^32^*E. coli* based bioluminescent arsenic sensors at 3.8 ppb^15^ and 5 ppb,^12^ a *B. subtilis* arsenic sensor at 5.9 ppb,^19^ and a *Pseudomonas fluorescens* arsenic sensor with a detection limit of 1 ppb.^33^ It is worth noting that the LODs for these arsenic sensors mentioned in prior works were estimated using least-squares regression, a widely accepted practice in sensor development. However, live bacterial cells exhibit substantial cell-to-cell variation, making statistical significance calculated with biological replicates a crucial metric for accurately determining the LOD. Therefore, we report our LOD at 0.01 μM (0.77 ppb) here with 95% confidence derived from 17 biological replicates.

### Characterization of Arsenic Whole-Cell Biosensor with *B. megaterium* Spores

An advantage of using *Bacillus* strains as chassis for whole-cell biosensors is their ability to sporulate. These spores are metabolically dormant and can therefore survive for long periods of time without nutrients and/or water in extreme environments. Once nutrients are available, these spores can rapidly germinate, becoming metabolically active vegetative cells. This characteristic, in the context of microbial biosensors, prolongs shelf life, removes cold chain considerations, and widens the scope of deployment for these sensors.^18^ This advantage is the key in designing sensors for point-of-need use, especially in austere environments, where preservation capabilities are minimal.

**Figure 3:**
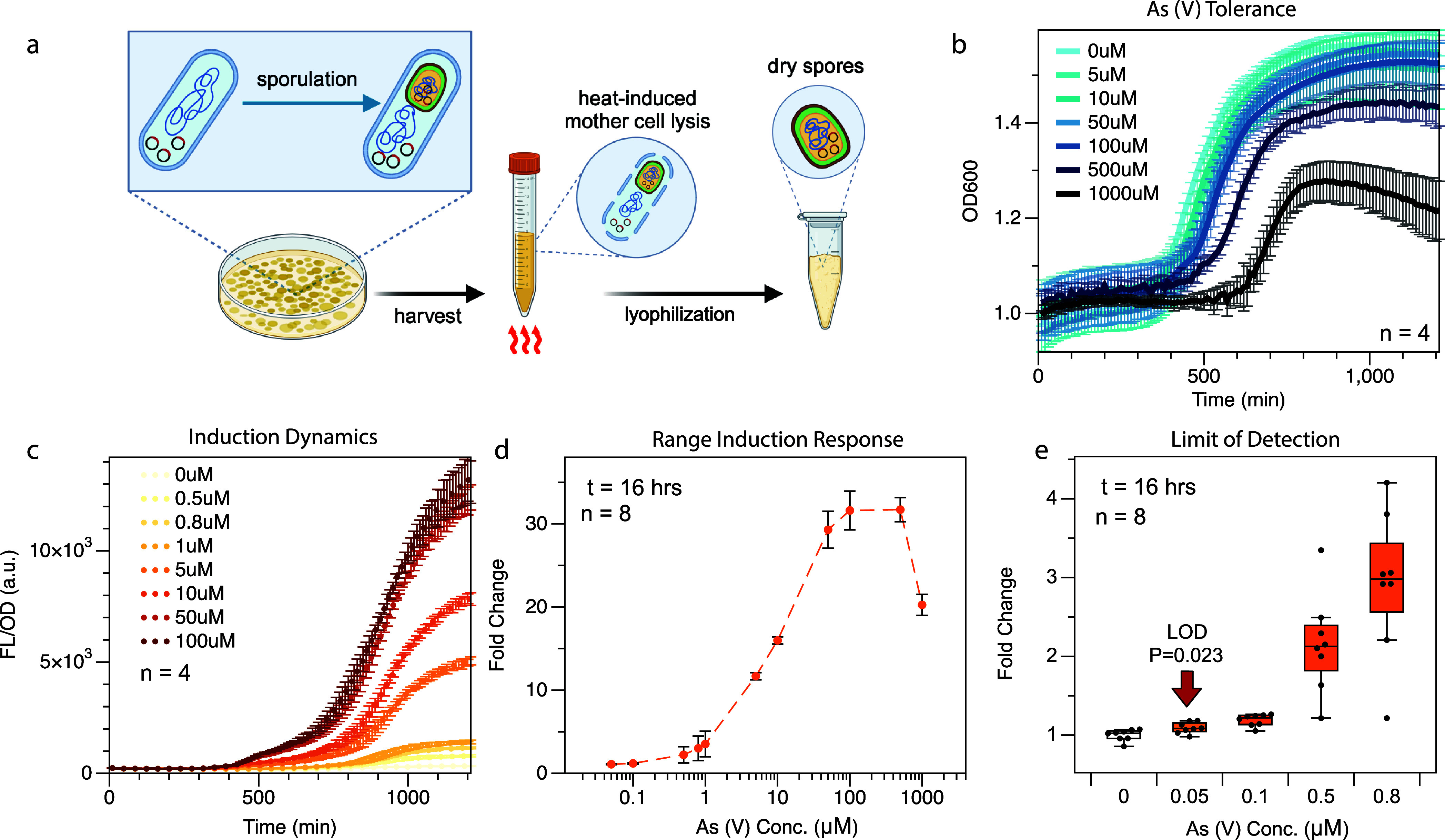
Sensor characterization with spores. (a) Sporulation and spore collection procedure. Illustration created with Biorender.com (b) Sporulation dynamics exposed to high arsenic concentrations ranging from 5 μM to 1000 μM over 16 h. Spores were hydrated to OD = 1.0 in LB media at *t* = 0. (c) Fluorescent signal dynamics: mCherry fluorescence normalized by OD_600_ over 16 h, following hydration with LB media containing Arsenic ranging from 0.5 μM to 100 μM at time = 0 min. (d) Sensor response to arsenic: spore-based sensor response to As(V) ranged from 0.05 μM to 1 mM, normalized to the 0 μM induction culture, measured 16 h posthydration. (e) Limit of detection: Induction response of the sensor to low-range As(V) concentrations, spanning from 0.05 μM to 0.8 μM. Plot shows statistical significance (*p* < 0.05) in sensor activation with as low as 0.05 μM. The number of technical replicates (*n*) used in each experiment is indicated in all plots, as spores were harvested in bulk.

We first generated the spores of our engineered *B. megaterium*. As illustrated in Figure 3a, we used a modified procedure based on the protocol published by Periago et al.^34^ to induce sporulation. Following sporulation, we scraped off the mixture of vegetative cells and spores. This mixture was subjected to high heat to eliminate all vegetative cells, leaving only spores. We divided the spores into two groups: fresh and lyophilized spores and were subjected to arsenic inductions directly without pregermination.

Then, we tested whether *B. megaterium* spores can germinate in arsenic-containing solution and perform sensing simultaneously. To achieve this, we back diluted fresh spores to an OD_600_ value of 1.0 in PBS and hydrated the cultures with deionized water containing a range of arsenic concentrations (0 μM to 1 mM), supplemented with 10× LB media. The growth profile, shown in Figure S2a, demonstrates that the time required to achieve significant germination (defined as reaching an OD_600_ of 1.1) varies depending on the arsenic concentration used for induction: 350 min for 0 μM and 5 μM; 420 min for 10 μM and 50 μM; 470 min for 100 μM; 520 min for 500 μM; and nearly 800 min for 1 mM. This result suggests that in comparison to vegetative cell proliferation, spore germination is less resistant to arsenic toxicity. Nevertheless, despite the delay in germination, the fluorescence signal increases along with As(V) concentrations, up to 200 μM (Figure S2b), indicating appropriate sensor performance up to this concentration. We also obtained the induction response profiles at low concentration of As(V), the LOD for arsenic was established at 0.05 μM, as shown in Figure S2c.

We repeated the experiment with lyophilized spores stored at room temperature for 3 weeks. The results, as shown in Figure 3b,c, indicated that it took approximately 440 min for cultures exposed to 5 μM to 100 μM arsenic to germinate (reach OD_600_ = 1.1), while for 500 μM arsenic it took 500 min, and for 1 mM arsenic it took 660 min. Although lyophilized spores showed a slight delay in germination compared to fresh spores, the presence of arsenic up to 100 μM did not significantly impact germination time. After 16 h, the fluorescence and OD_600_ signals were sufficiently distinct to differentiate concentrations up to 100 μM (Figure 3d), maintained the LOD at 0.05 μM (Figure 3e).

Our findings demonstrate that *B. megaterium* spores can serve as a robust, durable chassis for biosensors, functioning effectively even under conditions where germination may be inhibited, such as in the presence of arsenic. Unlike previous studies, where spores of engineered bacteria were rehydrated and germinated into vegetative cells for sensing,^19^ our work directly employs spores for arsenic detection. This approach eliminates the need for an extra germination step, showcasing the resilience of *B. megaterium* spores in directly sensing toxic compounds. While the LOD of spores is higher, indicating reduced sensitivity, and their arsenic tolerance is lower compared to vegetative cells, they offer substantial advantages in terms of storage flexibility and ease of transportation. Moreover, lyophilization and room temperature storage do not impair the spores’ sensitivity to As(V), reinforcing their potential in scenarios where portability outweigh the need for maximum sensitivity or rapid response time.

### Integrating the Whole-Cell Sensor with CMOS for Arsenic Detection

To fully realize the potential of the spore-based sensor for point-of-need applications, the fluorescent readouts must be measurable without relying on bulky and expensive laboratory instruments. Here, we integrated our biosensor in both *B. megaterium* in the forms of vegetative cells and spores with CMOS chip from our previous work.^23^ This 65 nm CMOS chip implements a 600–700 nm range on-chip bandpass optical filter, photodiodes, and processing circuitry, making it capable of reading both the OD_600_ and the mCherry fluorescent protein (Ex/Em: 587 nm/610 nm) when using a 550 nm-LED as the excitation light source.

**Figure 4:**
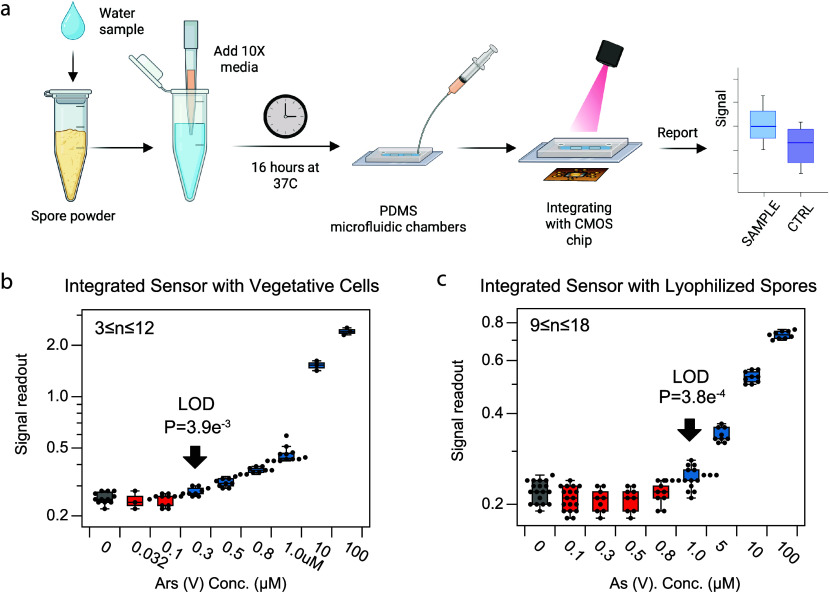
Integrated arsenic sensor characterization. (a) Illustration integrated arsenic sensor detection operation flow, partially created with Biorender.com. (b) Limit of detection when integrating CMOS chip with vegetative cells: Induction response of sensor to As(V) concentrations, ranging from 0.032 μM to 100 μM. Plot shows statistical significance in sensor activation with as low as 0.3 μM. *n* represents the number of biological replicates. (c) Limit of detection when integrating CMOS chip with lyophilized spores: Induction response of sensor to As(V) concentrations, ranging from 0.1 μM to 100 μM. Plot shows statistical significance in sensor activation with as low as 1.0 μM. *n* represents technical replicates, as spores were harvested in bulk.

For performance evaluation, we tested the sensor’s response using vegetative cells as a reference. This setup follows the same procedure as shown in Figure 2, where fresh *B. megaterium* cells were cultured for 16 h at 37 °C post arsenic series induction ranging from 0.032 μM to 100 μM. The mCherry fluorescent response was measured with our CMOS chip in two independent experiments, each with 3–12 replicates. As shown in Figure 4b, the integrated sensor demonstrated a LOD of 0.3 μM. Compared to the response shown in Figure 2e, where a commercial plate reader with excitation at 587 nm and emission at 610 nm achieved an LOD of 0.01 μM, the reduction in sensitivity in our system is notable. Nonetheless, ongoing advancements in CMOS technology are expected to improve the sensitivity of our system, bridging the gap with traditional laboratory instruments.

Subsequently, we integrated the CMOS chip with lyophilized spores to simulate a field-testing scenario. As depicted in Figure 4a, we added 900 μL of As(V)-contaminated water sample and 100 μL of 10× LB nutrient into lyophilized spores stored for over 6 weeks at room temperature. The spores were incubated for 16 h at 37 °C posthydration before measurement with the CMOS chip. The system was titrated with arsenic from 0.1 to 100 μL, in each condition, 9 to 18 measurements as technical replicates were obtained. Through two-tailed *t* test, we found that the arsenic LOD with this setup is 1.0 μM.

As presented in Table 1, the five versions of arsenic sensors we have tested are relevant in their corresponding potential application scenarios. For drinking water, both the EPA and WHO set the acceptable limit for arsenic in drinking water at 10 ppb, which corresponds to approximately 0.13 μM of arsenic. This makes the sensitivity of our *B. megaterium* sensor in both vegetative cells and spore forms, within application range. For soil arsenic content, the regulation limits vary drastically from country to country. Utilizing established arsenic extraction methods from soil and sediments, such as a combination of phosphate solution and microwave, achieves about 80% arsenic recovery.^35^ This method involves extracting 1 g of soil into 20 mL of phosphate buffered solution,^35^ leading to a LOD of 1.875 mg/kg of arsenic in soil. This is under the guideline limit by 19 states in the U.S. and most international guidelines, including Finland at 5 mg/kg, Canada at 12 mg/kg and UK at 32 mg/kg, etc.^3^ In theory, the sensitivity of our integrated sensor (CMOS chip + lyophilized spores) is applicable in testing arsenic content in soil using current inorganic arsenic extraction methods by most international standards and some US state standards. To achieve the USEPA’s SSLs for residential areas at 0.39 mg/kg,^3^ the sensitivity of our integrated sensor needs to be further improved. To test arsenic content in rice, various extraction methods achieve a recovery efficiency ranging from 75% to 100%.^36^ Using a setup of 1 g of rice and 20 mL of extracting solution, to meet the FDA’s recommendation of 0.1 mg/kg of inorganic arsenic in rice cereals for infants,^5^ the sensor’s LOD needs to be as low as 0.05 μM at a 75% recovery rate. In air monitoring, (OSHA) has set the permissible exposure limit (PEL) for inorganic arsenic at 10 μg/m^3^ in air, averaged over an 8-h workday. OSHA’s midget air bubbler sampling method, which bubbles up to 120 L of air into 10 mL of solution at a rate of 1 L per minute.^37^ Using the midget air bubbler to sample air that contains 10 μg/m^3^ of arsenic, will take 75 min to sample 75 L of air to achieve solution concentration of 1 μM. This means that the integrated sensor is theoretically applicable for field testing of airborne arsenic using a standard midget air bubbler.

**Table 1:**
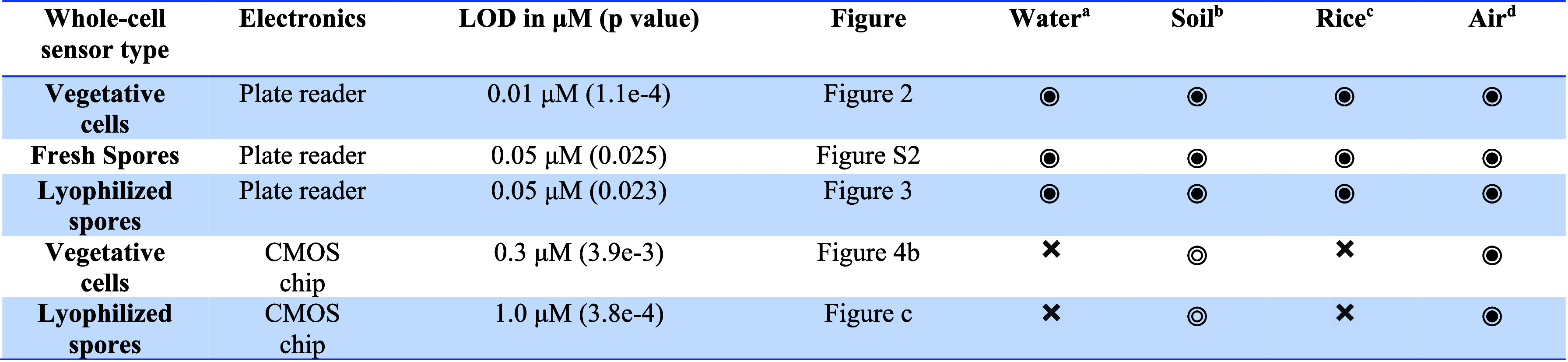
Limits of Detection of All Versions of Arsenic Sensors and Their Potential Applicable Scenarios^a^^,^^b^^,^^c^^,^^d^

a Based on EPA’s standard for drinking water, at 10 ppb.

b Based on EPA’s SSL for residential areas at 0.39 mg/kg (solid circle) and most international standards between 5 and 15 mg/kg (hollow circle).

c Based on FDA’s arsenic limit at 0.1 mg/kg in rice cereals for infants.

d Based on OSHA’s PEL of 10 μg/m^3^ of arsenic in air. 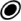 applicable, 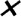 not applicable, 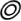 partially applicable.

While these calculations suggest that the sensor’s sensitivity is within range for various applications, this study was conducted using deionized (DI) water spiked with arsenic rather than real-world matrices such as groundwater, soil extracts, or food samples. Real environmental and biological samples introduce additional complexities, including potential interferences, pH variability, and matrix effects, which could impact sensor performance. Furthermore, direct validation in soil, food, or air samples was not performed in this study. Future work will focus on testing the biosensor in complex environmental samples to assess its robustness, accuracy, and potential need for sample pretreatment before real-world deployment.

## Conclusions

In this work, we successfully demonstrated the potential of *B. megaterium* as a chassis for developing whole-cell biosensors. By harnessing the natural resilience and sporulation capabilities of *B. megaterium*, we created a sensitive and portable arsenic detection system integrated with CMOS technology. The whole-cell sensor performed effectively in both vegetative cell and spore forms, with detection limits of 0.01 μM and 0.05 μM, respectively, well below the EPA’s arsenic limit for drinking water. Our reported LOD of 0.01 μM (0.77 ppb) for vegetative cells is among the lowest achieved for arsenic biosensors to date, confirmed with 95% confidence from 17 biological replicates. This surpasses other systems, including *E. coli* and *Bacillus subtilis*-based arsenic sensors. The integration with CMOS chips lays the foundation for the use of biosensors in portable and field-deployable applications, providing a significant reduction in equipment size and cost compared to traditional methods. The CMOS-based sensor maintained a detection limit of 0.3 μM with vegetative cells and 1.0 μM with spores, suitable for various environmental monitoring scenarios, including soil and air arsenic contamination.

This work demonstrates the unique capability of *B. megaterium* spores to detect arsenic directly, without requiring a germination step, even in arsenic’s toxic presence. This capability underscores the resilience of the *B. megaterium* spores, which can sense arsenic in conditions that inhibit germination. These attributes—remarkable resilience and ease of transport—gives potential for the sensor to be well-suited for resource-limited settings. The successful integration of biological and electronic systems in this study holds significant promise for the deployment of robust, cost-effective, and environmental monitoring solutions.

Future enhancements in the sensitivity of the integrated sensor could broaden its applicability, ensuring compliance with stringent regulatory standards for arsenic detection in diverse environments. Such improvements could be achieved by enhancing the sensitivity of the CMOS technology, which includes increasing the active area of photodetectors and optical filters to collect more photons/signals, redesigning the on-chip optical filters to better match the target wavelengths, and increasing the bias current of the front-end to reduce noise at the expense of slightly higher power consumption. Additional future work includes conducting field tests and calibrating the sensor with various samplers to standardize the procedure for different applications.

## Materials and Methods

### Construction of Arsenic Sensors

The *arsR* gene and *pArs*, *yknW*, *aprE*, *metA*, and *yngC* promoters, derived from *B. subilitis*,^38^ were synthesized by IDT. The pMM1522 vector (Mobitech) was linearized by PCR-amplification (Forward primer: 5′ cattactcgcatccattctcaggctgtctcgtctcgtctcatgcgcaaaccaacc 3′, Reverse primer: 5′ gcttggattctgcgtttgtttccgtctacgaactcccagcttaagtgaacgcaaaggtta 3′). Parts were assembled, along with the GFP gene, using overlapping UNSs^39^ and NEBuilder (New England Bio Laboratories). Cloning was carried out in *E. coli* DH10B cells. Plasmids were purified using a Qiagen QIAprep Spin Miniprep Kit (Qiagen 27104). Linear fragments were gel extracted and purified using MinElute Gel Extraction Kit (Qiagen 28606). Part sequences and plasmid maps are provided in Table S2. Confirmed DNA plasmids were transformed into *B. megaterium* protoplasts based on method previously established,^26,40^ detailed protocol is also included in the Supporting Information.

### Bulk Fluorescent Experiments

Bulk fluorescence measurement was employed to evaluate the expression of fluorescent proteins following chemical induction. For vegetative cells, overnight liquid cultures were initiated from freshly isolated colonies on LB agar plates, and then incubated in LB medium at 37 °C with shaking at 220 rpm for 16 h. Subsequently, a 200 μL subculture was established in a 96-well culture block by combining 196 μL of LB medium with antibiotics and 4 μL of the overnight culture. This subculture was grown for 4 h, then diluted 20-fold with LB medium containing the appropriate antibiotics (Tetracycline) and arsenic inducers in Na_2_HAsO_4_·7H_2_O (CAS 10048-95-0). The diluted cultures were incubated in a 96-well optic plate using a BioTek Synergy H1 plate reader for 16 h. The plate reader maintains conditions at 37 °C with maximum linear shaking, measuring fluorescence (GFP - Ex/Em: 485 nm/528 nm or mCherry-Ex/Em: 587 nm/610 nm) and OD_600_ every 10 min. For lyophilized spores, a mixture of 180 μL of arsenic-containing deionized water and 20 μL of 10× LB medium was used to rehydrate the spores to an OD of 1.0. These rehydrated spores were then grown in a 96-well plate in the BioTek Synergy H1 plate reader for 24 h under the same conditions, with measurements of fluorescence (mCherry-Ex/Em: 587 nm/610 nm) and optical density OD_600_ taken every 10 min.

### As(V) Tolerance Assay

*B. subtilis* and *B. megaterium* tolerance to As(V) were assessed on a dose–response curve. Overnight cultures were diluted to OD_600_ = 0.01 and were grown until OD_600_= 0.6; split into 2 mL aliquots and exposed to a range of As(V) concentrations (final concentrations: 0 mM, 0.5 mM, 1 mM, 2 mM, 5 mM) for 16 h with shaking at 37 °C. After exposure, 1 mL of each concentration was harvested via centrifugation, washed with PBS (Fisher; BP2944100) with a final resuspension of 0.5 mL. Viability was analyzed following manufacturer instructions using *Bac*Light Bacterial Viability Kit (Invitrogen; L7012). Survival was determined by a change in normalized fluorescence compared to 0 mM of As(V) exposure using BioTek Synergy H1). Confocal microscopy was performed by immobilizing samples on 2% agarose pads (Fisher; BP1356–500) using SP8 (Leica). Images were acquired and processed with Leica Application Suite software. Fluorescence was measured for SYTO9 (Ex/Em: 480 nm/500 nm) and Propidium Iodide (Ex/Em: 490 nm/635 nm). The sensitivity assay had three independent experiments in triplicate performed.

### Preparation of Spores from *Bacillus megaterium*

Cells were initially cultured from a single colony in 5 mL of LB medium at 37 °C, agitated at 220 rpm overnight. Following this, 200 μL aliquots were evenly distributed onto spore-forming media plates, prepared with 5 g tryptone, 2.5 g yeast extract, 1 g glucose, 15 g agar, and deionized water up to 1 L, with the pH adjusted to 7.0, with appropriate antibiotics. These plates were then incubated at 30 °C for a duration of 5 days. Subsequently, the grown biomass was scraped off from the plates and resuspended in deionized water in 15 mL conical tubes, with the suspension diluted to achieve an OD_600_ of 1.0. To ensure the inactivation of vegetative cells, the tubes containing the suspension were placed in a water bath at 80 °C for 10 min, then allowed to cool at 20 °C for another 10 min. The final step involved lyophilizing the suspension within the same 15 mL conical tubes, preparing it for further analysis or storage. Lyophilized spores were stored in room temperature.

### Statistical Analysis

Fluorescence per optical density (FL/OD) signals were adjusted by subtracting the background, which was determined using the average autofluorescence signal derived from the FL/OD measurements of three colonies of nontransformed cells. Data analysis was conducted using Microsoft Excel. The sample size was not predetermined through statistical methods. All single colonies were selected randomly from agar plates following the transformation of cells with purified circular DNA, employing antibiotics as selection markers. No manual methods for group allocation were implemented. Each plate originated from a distinct transformation event, and all colonies on a plate were considered biological replicates. Throughout the experiments, no data were excluded from analysis.

The LOD was determined statistically. For vegetative cells, we calculated the signal fold changes for each colony by dividing the fluorescent output of an induced culture by the fluorescent output of the uninduced culture. For spores, the signal fold changes for each measurement were calculated by dividing the fluorescent output of an induced culture by the average fluorescent output of the uninduced culture. The sensor’s LOD was established when the fold changes at a given concentration were significantly higher than those of the uninduced group, based on a two-tailed *t* test with *p* < 0.05. For CMOS experiments, signals were analyzed without fold activation calculation, but the same statistical tests and significance thresholds were used to determine the LOD.

## Supporting Information Available

Additional experimental results including promoter characterization, growth curves in different media, GFP expression dynamics, and arsenic tolerance assays with microscopy images (Figure S1); characterization of the arsenic sensor using fresh spores is detailed, including sporulation under arsenic exposure, dose–response curves, and determination of the limit of detection (Figure S2); a full protocol for protoplast transformation of *Bacillus megaterium* is included, along with recipes for all required media and buffers; finally, DNA sequences for all plasmid constructs and sensor components used in this study are listed, including the pMM1522 backbone, arsenic-responsive elements (pArs, pArs-ArsR), sfGFP, and alternative promoters (PDF)
